# Discovering, Indexing and Interlinking Information Resources

**DOI:** 10.12688/f1000research.6848.2

**Published:** 2015-11-17

**Authors:** Fabrizio Celli, Johannes Keizer, Yves Jaques, Stasinos Konstantopoulos, Dušan Vudragović

**Affiliations:** 1 Food and Agriculture Organization of the UN, Rome, Italy; 2 NCSR Demokritos, Athens, Greece; 3 Institute of Physics Belgrade, University of Belgrade, Belgrade, Serbia

**Keywords:** Linked Data, Text Categorization, Recommender Systems, Web Crawling, AGRIS, SemaGrow

## Abstract

The social media revolution is having a dramatic effect on the world of scientific publication. Scientists now publish their research interests, theories and outcomes across numerous channels, including personal blogs and other thematic web spaces where ideas, activities and partial results are discussed. Accordingly, information systems that facilitate access to scientific literature must learn to cope with this valuable and varied data, evolving to make this research easily discoverable and available to end users. In this paper we describe the incremental process of discovering web resources in the domain of agricultural science and technology. Making use of Linked Open Data methodologies, we interlink a wide array of custom-crawled resources with the AGRIS bibliographic database in order to enrich the user experience of the AGRIS website. We also discuss the SemaGrow Stack, a query federation and data integration infrastructure used to estimate the semantic distance between crawled web resources and AGRIS.

## Introduction

AGRIS ( http://agris.fao.org/) is the International System for Agricultural Science and Technology, a collection of nearly 8 million multilingual bibliographic resources spanning the last forty years and produced by a network of more than 150 institutions from 65 countries. AGRIS is currently part of the CIARD initiative ( http://www.ciard.net/), a self-described “global movement dedicated to open agricultural knowledge”. Some AGRIS data sources are unique ( http://aims.fao.org/activity/blog/agris-enriched-data-fao) to the system and AGRIS is the only way in which they can be accessed. The system’s goal is to make agricultural research globally discoverable, and as evidenced by Google Analytics it supports both developed and developing countries. Indeed, AGRIS is accessed from more than 200 countries and territories, reaching peaks of 250,000 visits per month. AGRIS users belong to two very different categories: the general public and agriculture professionals. In particular, a survey conducted at the end of 2014 helped to better describe the AGRIS audience [ Celli *et al.*, 2015]: researchers, professors, and graduate students looking for bibliographies, librarians, cataloguers, and people responsible for managing and disseminating research outcomes to the community and the rest of the world (including small and big journal publishers), and government officers asking for reports on specific topics. Since December 2013, AGRIS adopted a LOD (Linked Open Data) infrastructure [ Anibaldi *et al.*, 2015], which allowed the creation of mashup pages, where users looking for specific topics (e.g. impacts of climate change in a country) can access a publication from the AGRIS database, combined with other related resources extracted from other preselected datasets. External resources available in AGRIS mashup pages are not only bibliographic metadata, but also distribution maps, statistics, germplasm accessions, and so on. In this paper we explore a new data source available in AGRIS mashup pages: the web itself.

Nowadays, scientists and researchers publish their results not only in journals or at conferences, but also via web 2.0 tools and other media [ Kouper, 2010; Shema *et al.*, 2012] in order to efficiently and broadly communicate their outcomes; this technique also helps scientific research reach the general public, since newspapers, magazines and science blogs are often the quickest way to reach people informally. Blogs and other websites may also contain a corpora of ongoing research activities, unpublished material, grey literature, quick discussions, and experiments with negative results and ideas. The problem is that this information is usually not exposed using web services that can be consumed by machines, and the only way to access this rich amount of data is to use web search engines that typically return thousands of results, largely meaningless. In addition, most blogs and websites are not well categorized and so it is difficult for users and machines to discover what is actually relevant to the topic of interest.

In this context, we believe that it is important for AGRIS users – especially for researchers – to have access to those valuable pieces of information that are neither exposed in a database nor accessible via web service. Our goal is to crawl the web, starting from a list of manually preselected websites and then apply a set of machine learning algorithms to categorize discovered web resources. In recent years, much research has been done to crawl and mine the web. Numerous solutions have been proposed [ Devi *et al.*, 2015; Liakos *et al.*, 2015; Soulemane *et al.*, 2012] to cope with the size of both the publicly indexable and the hidden web employing added semantics to discovered resources and to reuse them in fact sheets and mashups. In fact, it is not only important to discover web links, but also to process them in a way that allows reuse in multiple scenarios. The adoption of ontologies and LOD methodologies helps the analysis and enrichment of discovered web resources [ Berendt *et al.*, 2004]. Our work shows how it is possible to apply semantic enrichment to crawled web resources and to use this semantic knowledge to enhance the AGRIS web portal. More specifically, our work leverages Semantic Web technologies and the knowledge encoded in the AGROVOC ( http://aims.fao.org/standards/agrovoc/about) thesaurus in order to recommend web resources that are relevant to a given AGRIS bibliographic item. AGROVOC is a multilingual vocabulary containing more than 32,000 agricultural concepts in 22 languages, aligned with 16 other multilingual knowledge organization systems related to agriculture, and developed by FAO over the course of thirty years [ Caracciolo *et al.*, 2011]. Adopting Semantic Web and LOD best practices and technologies, AGROVOC vocabulary items have been assigned URIs, organized into a SKOS-XL concept scheme ( http://www.w3.org/TR/skos-reference/skos-xl.html), and served both as Linked Open data and via SPARQL endpoint ( http://www.w3.org/TR/sparql11-overview).

In this article we discuss crawling and analysing web resources to populate our “Crawler Database”; a SPARQL endpoint with AGROVOC annotations of web resources identified by the URL from which they were crawled. By providing web resources with semantics we can use the AGROVOC descriptions of AGRIS bibliographic entries to interlink AGRIS and the Crawler Database. This linking is then exploited by a recommender that identifies web resources that are relevant to AGRIS entries. Furthermore, we also discuss the preliminary testing of the SemaGrow Stack ( http://www.semagrow.eu/) as the computational infrastructure for interlinking the AGRIS bibliographic database with the Crawler Database. The *query federation* and *data integration* functionalities of the SemaGrow Stack facilitate setting up experiments aiming at estimating semantic similarity between AGRIS entries and other resources. Although the core example that we discuss in this paper is based on the entities described in the Crawler Database, the power of the SemaGrow Stack is that it allows the re-use of this software in the context of different mashup pages combining AGRIS with a variety of LOD sources.

The entire process we discuss in this paper has already been implemented and integrated in the AGRIS website. While the tuning of the recommender system to compute accurate similarities is still an ongoing process, AGRIS mashup pages are enriched with the content of the Crawler Database and statistics are being collected in order to train the recommender system using user behaviour. In addition, the workflow and the components described in this paper can be used in any domain, so they are not restricted to agriculture; one can simply use another thesaurus to annotate web resources and populate the Crawler Database.

## Crawling and indexing the web

**Figure 1.**
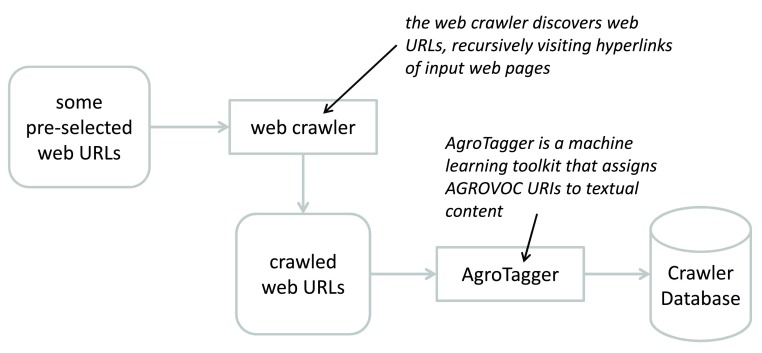
The process of crawling and indexing the web.

The process of discovering and tagging web resources to display new content in the AGRIS website is based on two backend components: a customized Apache Nutch web crawler and AgroTagger. Figure 1 provides an overview of the entire process. As a starting point, in order to display relevant content in AGRIS, we manually preselect related websites to be used as input for the web crawler. Using these URLs the web crawler discovers other related web URLs, while AgroTagger assigns AGROVOC URIs to web URLs and creates the Crawler Database. In the next two sections we describe the two backend components.

### The web crawler

**Figure 2.**
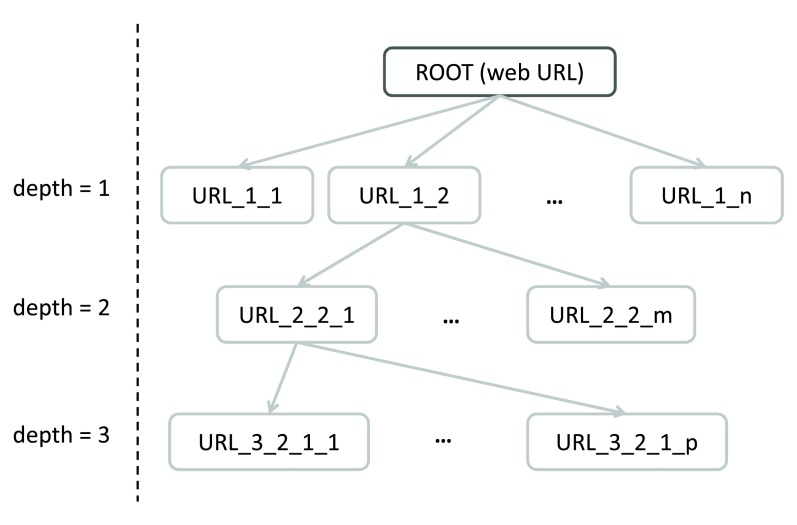
Execution of the web crawler using depth=3.

A web crawler is a piece of software that methodically and automatically analyses web pages provided as input. Each input web page is a ROOT of the crawling process. During the analysis of a web page, the web crawler discovers all the hyperlinks available in that page, adding them to the list of web pages to be visited. The process stops at a specific *depth*, i.e. the number of hops a discovered link is from the ROOT. The depth parameter is defined by the user of the web crawler, with the idea that links decrease in relevance as their distance from the ROOT grows. At the end of the crawling process, a list with discovered web URLs is produced. Figure 2 shows an example execution of the web crawler.

In order to implement the process of enriching the AGRIS website with relevant web resources, we used a customized version of Apache Nutch ( http://nutch.apache.org/), a highly extensible, scalable and configurable open-source web crawler. Web URLs provided as input were manually selected by experts in the domain of agriculture, in order to start the process from “trusted” and valuable websites. Currently, the depth parameter is set to *5*, since this value seems to be a good compromise between discovering a good quantity of relevant resources and completing the task within an acceptable response time. The application, including the customized Apache Nutch web crawler and some Bash scripts to run it is available on GitHub ( https://github.com/fcproj/agrotagger/tree/master/crawler). The application requires three input parameters:

- The depth of the crawling process
- The path to the directory that stores the output of the process
- The path to the text file that contains the list of web URLs used as ROOTs by the crawler

The output of the application is a text file containing a structured list of discovered web URLs. Such file is built reading segments generated by the Apache Nutch crawler, using the “ readseg –dump” command line tool, as documented in the Apache Nutch documentation ( http://wiki.apache.org/nutch/bin/nutch_readseg). The file structure is very easy to learn: a text file containing a URL per line; the URL can be specified after the “ URL::” tag; otherwise, if the URL was discovered in an anchor, it can be specified in the “outlink: toUrl:” tag (if the anchor has a name, it is reported after the “anchor:” tag). Here is a snapshot of the output file:



                        URL:: http://%20www.umabroad.umn.edu/students/healthsafety/emergency.php
URL:: http://10-29-2013-tfic-luncheon.eventbrite.com/
URL:: http://1z8jbr3nz90837simd2d2fwoktj.wpengine.netdna-cdn.com/wp-content/uploads/2014/05/Nina-Hale-Inc-FactSheet.pdf
URL:: http://2014.northernspark.org/
URL:: http://2014.northernspark.org/project/chimera
  outlink: toUrl: http://media2.northernspark.org/wp-includes/wlwmanifest.xml anchor:
  outlink: toUrl: http://2014.northernspark.org/partners/arts-culture-and-the-creative-economy-program-of-the-city-of-minneapolis anchor:
  outlink: toUrl: http://2014.northernspark.org/project/bell-museum-staff anchor:
URL:: http://aaea.execinc.com/edibo/JobMarketCandidates
  outlink: toUrl: http://www.aaea.org/ anchor: AAEA
  outlink: toUrl: http://aaea.execinc.com/edibo/LoginHelp anchor: Create an Account / Need Help Logging In
  outlink: toUrl: http://www.aaea.org/about-aaea/aaea-sections anchor: AAEA Sections
                    


### AgroTagger classifier

AgroTagger engine ( https://github.com/fcproj/agrotagger) is a toolkit that assigns semantic terms to textual content. At a high level of abstraction, it can be considered as a keyword extractor that uses the AGROVOC thesaurus to extract keywords from a set of web URLs. It is based on MAUI ( https://code.google.com/p/maui-indexer), a tool that combines a keyphrase extraction algorithm and a machine learning toolkit for the identification of topics in textual documents. AgroTagger currently works only with English documents; in fact, AgroTagger is using the MAUI model trained with 780 full-text documents using AGROVOC in English [ Medelyan & Witten, 2008]. Training MAUI with AGROVOC in other languages will allow the applicability of AgroTagger in a multilingual environment, even if further tests need to be performed to understand how the tool behaves with non-Latin characters. Regarding accuracy, a recent test (conducted outside the study proposed in this paper) in which AgroTagger results were analysed by professional indexers showed an accuracy of approximately 80%; in 20% of the cases the results were too broad. In brief, the accuracy measurement was carried out by domain experts actively involved in the development of the AGROVOC vocabulary based on manual annotations of a test sample from the AGRIS database. The test sample was composed of 32 documents already indexed by FAO professional cataloguers making use of the AGROVOC thesaurus; those documents were randomly selected from the AGRIS database, according with some constraints: the link to the full-text had to be available; they needed to be produced by FAO cataloguers; they needed to be indexed with at least 8 AGROVOC keywords. Then, AgroTagger was executed to automatically annotate such documents; lastly, keywords identified by cataloguers were compared with keywords assigned by AgroTagger, determining an accuracy of AgroTagger of around 80%.

**Figure 3.**
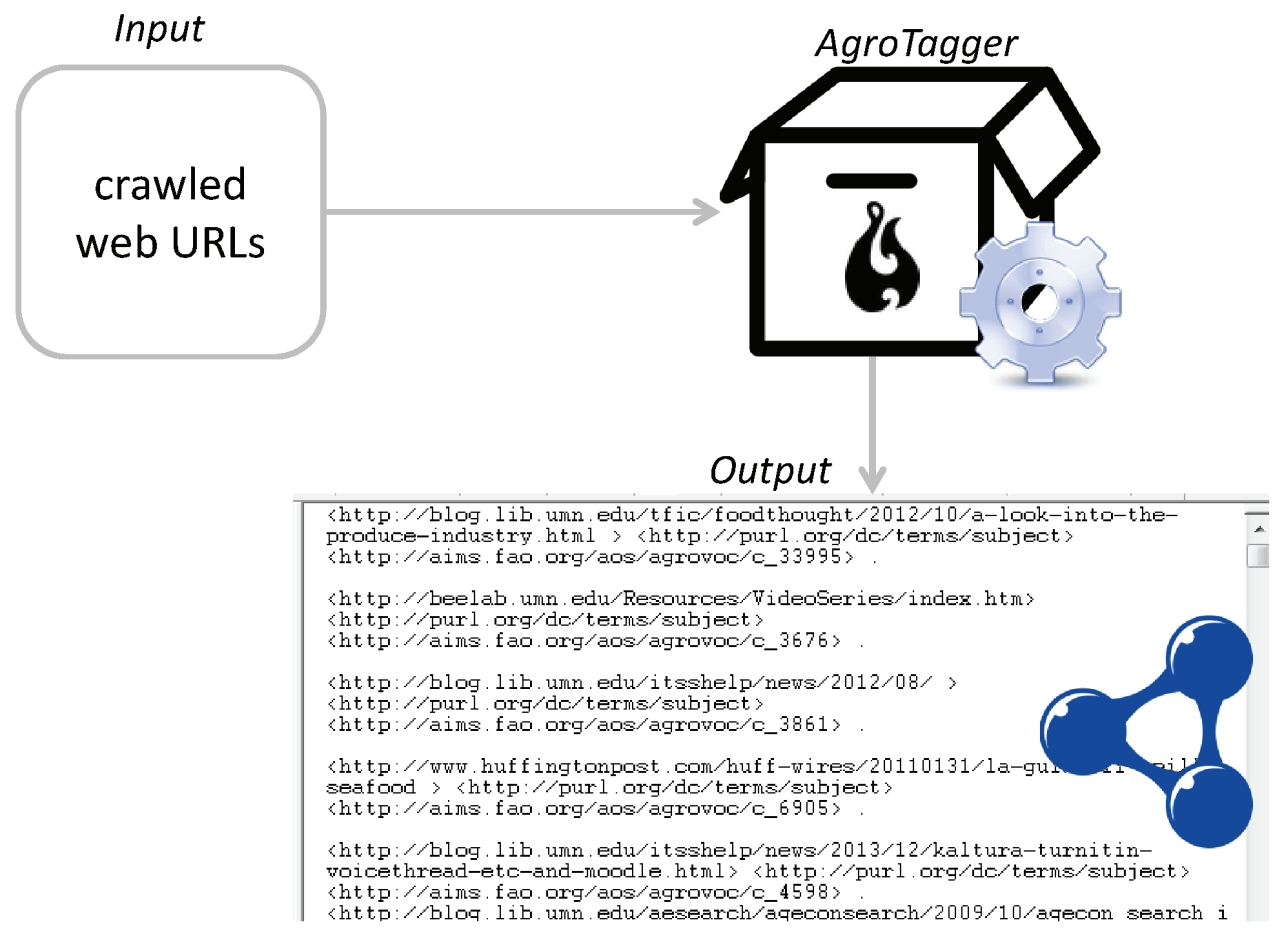
AgroTagger workflow.

In the process described in this paper, we apply AgroTagger to web URLs discovered by the web crawler and we annotate such URLs with AGROVOC URIs. Annotations are stored in a triple store (the Crawler Database) after which a recommender system defines some relevant combinations between AGRIS bibliographic resources and web documents, making use of AGROVOC as the backbone of the entire process. Considering AgroTagger as a black box (as depicted in Figure 3), we can describe its I/O as:

- Input: web URLs discovered by the web crawler
- Output: a set of triples that annotate web URLs

**Figure 4.**
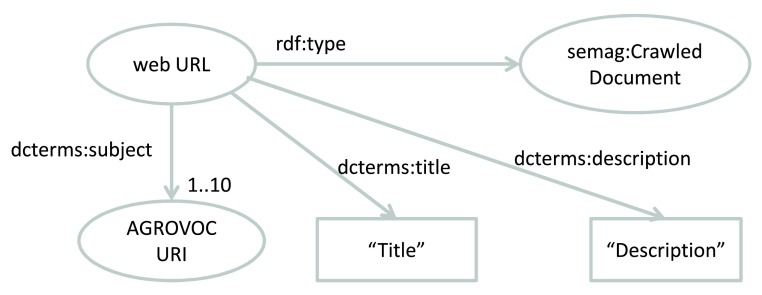
RDF schema of AgroTagger output annotations.

AgroTagger is a multi-threaded application, guaranteeing better performance while manipulating web URLs. For each web URL available in the input file, AgroTagger:

- Downloads the resource available at the given web URL and converts it to a text file
- Runs the MAUI indexer trained with AGROVOC
- Produces a set of annotations as RDF triples. The RDF schema of AgroTagger output annotations is shown in Figure 4)

Currently, AgroTagger produces an RDF NTRIPLE file that describes semantic annotations for web URLs. Here is an example of an annotated web URL:



                        <http://www.eje.cz/pdfs/eje/2008/04/01.pdf> <http://www.w3.org/1999/02/22-rdf-syntax-ns#type> <http://semagrow.eu/rdf#CrawledDocument> .
<http://www.eje.cz/pdfs/eje/2008/04/01.pdf> <http://purl.org/dc/terms/title> "Word Pro - Hoshizaki.lwp" .
<http://www.eje.cz/pdfs/eje/2008/04/01.pdf> <http://purl.org/dc/terms/subject> <http://aims.fao.org/aos/agrovoc/c_24778> .
<http://www.eje.cz/pdfs/eje/2008/04/01.pdf> <http://purl.org/dc/terms/subject> <http://aims.fao.org/aos/agrovoc/c_27496> .
<http://www.eje.cz/pdfs/eje/2008/04/01.pdf> <http://purl.org/dc/terms/subject> <http://aims.fao.org/aos/agrovoc/c_12332> .
                    


Training AgroTagger with a different thesaurus allows one to reuse the entire workflow and components described in this paper in completely different research domains.

## The SemaGrow Stack

Scalable, efficient, and robust data services are re-shaping the way that data analysis techniques are applied to the heterogeneous data cloud and enable data-intensive and inter-disciplinary approaches to science. SemaGrow is an FP7 European project developing such infrastructure services. The core technical outcome of the project is the SemaGrow Stack. The SemaGrow Stack implements a SPARQL endpoint that federates SPARQL endpoints, transparently optimizing federated queries and dynamically integrating heterogeneous data models by applying the appropriate vocabulary transformations to queries and results [ Charalambidis *et al.*, 2015]. There are several key features of the SemaGrow Stack that address AGRIS use cases: it provides a querying interface that uses the result of ontology alignment to completely hide schema heterogeneity and also applies methods from database research and artificial intelligence that take into account data contents and optimize federated querying plans. The ontology alignment and dynamic vocabulary transformation facilities allow us to take advantage of multiple agricultural knowledge organization systems that have been aligned to AGROVOC. In this manner, one can develop AGROVOC-aware applications and use them over non-AGROVOC (but aligned) datasets without modification. The query optimizer is based on methods that automatically extract detailed metadata about the content of the federated endpoints, overcoming the lack of detail in manually provided annotations [ Zoulis *et al.*, 2015]. As an added benefit, the SemaGrow Stack implements fail-over mechanisms that fall back to alternatives in the face endpoint unavailability. It furthermore does not require any modification of the federated endpoints or any other obtrusion of current workflows.

The SemaGrow Stack is developed and distributed as open-source software ( https://github.com/semagrow) and requires an Apache Tomcat environment ( http://tomcat.apache.org/) in order to be deployed and executed.

## The recommender system

The Crawler Database is composed of triples generated by AgroTagger. At this stage, the biggest problem is to compute a meaningful intersection with the AGRIS bibliographic database in order to display relevant information in an AGRIS mashup page. First of all we need to define the concept of “ *meaningful combinations*”. A naïve approach is based on counting the number of AGROVOC URIs in common between resources from the Crawler Database and resources from the AGRIS bibliographic database. Thus, for an AGRIS mashup page, we can state that we want to display those resources from the Crawler Database having as many AGROVOC URIs as possible in common with the AGRIS bibliographic entry. To implement this naïve algorithm we developed a recommender system ( https://github.com/fcproj/recommender), a JAVA component that computes meaningful combinations between the Crawler Database and the AGRIS database, and generates a new triplestore: the “Recommender Database”. The recommender system runs as required as new data is periodically generated by the web crawler.

**Figure 5.**
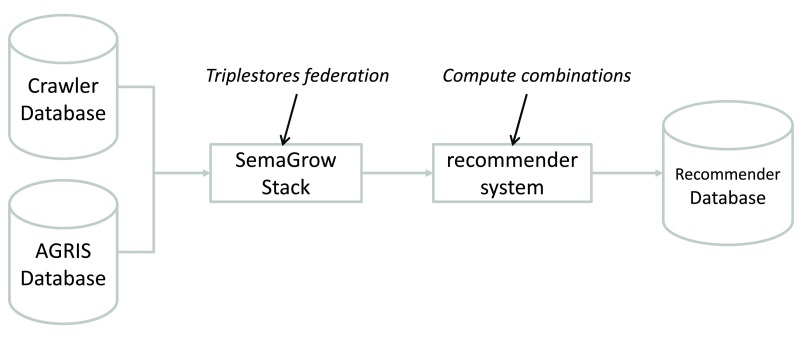
Usage of the SemaGrow Stack as intermediate layer for the generation of the Recommender Database, which contains meaningful combinations between the AGRIS bibliographic database and the Crawler Database.

The recommender system can make use of the SemaGrow Stack as the backbone of the process, as depicted in Figure 5: in this way, the recommender system is able to compute meaningful combinations between all datasets federated by SemaGrow. Combinations are computed by counting the number of common “dcterms:subject” URIs ( http://dublincore.org/documents/dcmi-terms/) between entities of the federated datasets (in the case of the AGRIS dataset, “dcterms:subject” URIs are AGROVOC URIs). The SemaGrow Stack is not strictly necessary, since the recommender system can also work querying two target SPARQL endpoints one by one. In any case, the SemaGrow Stack allows code reuse for all datasets; it is sufficient to configure the SemaGrow Stack by defining the URLs of the SPARQL endpoints included in the federation, and the recommender system can work using a single endpoint. Without the usage of the SemaGrow Stack as an intermediate layer, there is the need to modify the code to add further SPARQL endpoints to the process. Let’s explain this statement with an example. Currently, the recommender system has two ways of working (the user can configure it to use either mode): “federated”, which requires a single SPARQL endpoint, and “individual”, which accepts two SPARQL endpoints. The “federated” mode makes use of the SemaGrow Stack: if the user configures the Stack defining three or four endpoints in the federation, the recommender system can combine all of them. On the contrary, the “individual” mode works with a maximum of two SPARQL endpoints and would require additional software code if one wanted to add further endpoints to the process.

Our experiments focus on the usage of the recommender system to interlink the AGRIS database and the Crawler Database, storing computed combinations in the Recommender Database. At this stage, the algorithm is very easy: we simply need to count the number of common AGROVOC URIs (expressed as objects of a “dct:subject” predicate) between entities of the AGRIS dataset and the Crawler Database. Using the SemaGrow Stack and the single query “federated” mode, the SPARQL query to implement the algorithm is:



                    PREFIX dct: <http://purl.org/dc/terms/>
PREFIX rdf: <http://www.w3.org/1999/02/22-rdf-syntax-ns#>
SELECT distinct ?s (COUNT(distinct ?o) as ?NELEMENTS) WHERE {
  <$AGRIS_URI> dct:subject ?o .
  ?s dct:subject ?o .
  ?s rdf:type <http://semagrow.eu/rdf#CrawledDocument> .
}
GROUP BY ?s
ORDER BY DESC(?NELEMENTS)
LIMIT 20
                


This SPARQL query sorts resources from the Crawler Database by the number of AGROVOC URIs in common with a given AGRIS entity <$AGRIS_URI> after which it takes the 20 most relevant web resources and stores them in the Recommender Database as a set of recommendations for an AGRIS URI. In the query, the statement including the rdf:type predicate is only needed in order to get results from the Crawler Database; since the recommender system can work with any dataset federated by the SemaGrow Stack, the type needs to be an application-dependent parameter so that it can be configured to query different datasets.

Unfortunately, there are still few limitations with the SemaGrow Stack. First of all, SPARQL queries must be optimized and they are affected by their content. For instance, using a FILTER statement makes for a high response time. Thus, in order to identify entities from the different datasets federated by the SemaGrow Stack, it is necessary that such datasets define an “rdf:type” ( http://www.w3.org/TR/rdf-schema/) for their entities in order to avoid the usage of the FILTER statement. Moreover, given that at the time of these experiments the SemaGrow Stack distribution was limited and allowed only SPARQL queries with dct:subject and/or rdf:type predicates, we ran our experiments using the “individual” mode during processing and testing. At the time of publication, our final experiments using the “federated” mode are very promising; SPARQL queries that combine triples from federated datasets show a response time comparable to the execution time of a process that runs individual SPARQL queries to different endpoints and programmatically combines their results.

The “individual” mode that makes use of two different SPARQL queries to two different SPARQL endpoints: AGRIS and the Crawler Database. The first SPARQL query provides the list of AGROVOC URIs (“dct:subject” predicate) for a given <$AGRIS_URI>:



                    PREFIX dct:<http://purl.org/dc/terms/>
SELECT ?term WHERE {
   <$AGRIS_URI> dct:subject ?term .
}
                


For all AGROVOC URIs provided by the previous query, a second SPARQL query computes the crawled web URLs which contain that AGROVOC URI:



                    PREFIX dct:<http://purl.org/dc/terms/>
SELECT ?url WHERE {
   ?url dct:subject <$AGROVOC_URI>.
   ?url rdf:type <http://semagrow.eu/rdf#CrawledDocument> .
}
                


Then, a custom algorithm implemented in JAVA is used to count the number of common AGROVOC URIs between the AGRIS entity and web URLs returned by the previous query, storing the top 20 most relevant web resources in the Recommender Database.

### The similarity index

In the Recommender Database, recommendations for the same AGRIS URI are sorted by relevance (currently, the relevance is given by the custom algorithm calculated using the number of AGROVOC URIs in common with the AGRIS resource). The recommender system provides a *Similarity Score* for each recommended web URL, i.e. the percentage of similarity between an AGRIS resource and a recommended web URL. There are three variables to take into account in order to determine the Similarity Score:

- the number of AGROVOC URIs associated with an AGRIS record (we will refer to this variable as #AGRIS);
- the number of AGROVOC URIs associated with a recommended web URL ( #WEB) and
- the number of common AGROVOC URIs between the web resource and the AGRIS record ( #COMMON).

A naïve approach would consider the Similarity Score as the ratio given by the division between #COMMON and #AGRIS:

$$\text{Score}=\frac{\#\text{COMMON}}{\#\text{AGRIS}}$$

Unfortunately, this approach has some problems when the AGRIS record is associated with few AGROVOC URIs. For instance, consider the scenario described in Table 1.

**Table 1.** Example of naïve Similarity Score.

**#AGRIS**	10	10	10	6	6	3	3
**#WEB**	10	10	10	10	10	10	10
**#COMMON**	10	8	2	6	2	3	1
**Naïve Score**	1.0	0.8	0.2	1.0	0.33	**1.0**	**0.33**

The final two scores have an obvious problem of overestimation: the AGRIS record is associated with only 3 AGROVOC URIs and, even if the web URL has 3 common URIs with the AGRIS record, a score of 1.0 (100%) is too much to predict the similarity. Thus, we need an index to adjust the score when the AGRIS record has few AGROVOC URIs associated. This Similarity Index must be 1 in the case of maximum similarity, and 0 in the case of no common AGROVOC URIs. A naïve Similarity Index would be:

$$S=1-\frac{\#\text{WEB}-\#\text{COMMON}}{\#\text{WEB}}$$

The problem with this index is that # *WEB* is equal to 10 in most cases, since AgroTagger assigns 10 AGROVOC URIs to web resources crawled by the web crawler. Thus, *S* = 1 only if #AGRIS is 10 and #COMMON is 10, but this situation is highly improbable, since #AGRIS is on average equal to 6. If we define a threshold *τ* to determine when a number of common AGROVOC URIs is relevant to determine a good Similarity Index, we can improve the quality of the Similarity Score. Currently, we have defined *τ* equal to 6, which means that all cases where #COMMON is equal or bigger than 6, the Similarity Index must be 1.0. In this way, defining the correction factor *k*:

*k* = min( *τ*, # *COMMON*), *where τ* = 6

The Similarity Index *σ* can be computed as:

$$\sigma =1-\frac{\tau -k}{\tau }$$

This index has all the properties we are looking for: if #COMMON is 0, *σ* is 0; if #COMMON is equals or bigger than the threshold *τ* (6), *σ* is 1. At this point, we can redefine the Similarity Score as:

$$\text{Score}=\frac{\#\text{COMMON}}{\#\text{AGRIS}}\times \sigma$$

This approach further improves the Similarity Score. Since #WEB is never bigger than 10, while #AGRIS could be ideally any positive integer, we can modify the denominator of the first factor introducing the upper limit *T*, which is the maximum number of AGROVOC URIs associated to an AGRIS resource that is meaningful to the computation of the Similarity Score (we define *T* = 10):

$$\text{Score}=\frac{\#\text{COMMON}}{\min(T,\#\text{AGRIS})}\times \sigma =\frac{\#\text{COMMON}}{\min(T,\#\text{AGRIS})}\times (1-\frac{\tau -\min(\tau ,\#\text{COMMON})}{\tau })$$

Where: *T* = 10 and *τ* = 6.

Table 2 revises the scenario described in Table 1, computing the similarity score with the last formula.

**Table 2.** Revised example of Similarity Score.

**#AGRIS**	10	10	10	6	6	3	3
**#WEB**	10	10	10	10	10	10	10
**#COMMON**	10	8	2	6	2	3	1
**Naïve Score**	1.0	0.8	0.2	1.0	0.33	1.0	0.33
**Revised Score**	1.0	0.8	**0.07**	1.0	**0.11**	**0.5**	**0.06**

Considerations about the custom algorithm. The custom algorithm to compute meaningful combinations is an area for further research and improvement. In particular, other parameters may play a key role to define the relevance of a web resource. For instance, the system might check if some AGROVOC terms used by an AGRIS record appear in the title of the crawled web resource, or in its description. Then, AgroTagger could also be improved to return the ranking score of each AGROVOC URI assigned to a web resource, in order to use such a score in the algorithm that computes combinations. In addition to that, we can observe users' behaviour in an AGRIS mashup page in order to assign more relevance to web resources more frequently clicked by AGRIS users. Furthermore, interviews of users will be conducted to evaluate the relevance of resources displayed in AGRIS mashup pages. Finally, there may be other more exotic algorithms to calculate similarity that can give greater relevance to end users, but such experiments were outside the scope of this project.

### Analyzing the algorithm performance

The algorithm was executed on 775,297 AGRIS URIs; the crawler database was composed of 17,320,363 triples; the computation generated 19,738,145 triples for the recommender triple store. The recommender system ran in a CentOS 5 environment with the following configuration:

- **Processor:** Intel(R) Core(TM) i7 2.80GHz
- **Recommender system:** 2GB RAM on an 8GB RAM machine, hosted in Rome
- During the execution of the experiment, the “IPTraf” ( http://iptraf.seul.org/) tool computed a TCP flow rate of between 30 and 45 Kbits/s.

The two SPARQL endpoints to intersect were:

- **AGRIS SPARQL endpoint:** hosted in Malaysia, at MIMOS ( http://www.mimos.my/), configuration unknown
- **Crawler Database SPARQL endpoint**: hosted in Serbia, at IPB ( http://www.ipb.ac.rs/index.php/en/), configuration unknown

We ran two experiments. In a first experiment we used the SemaGrow Stack as the backbone of the process, so the recommender system worked in the “federated” mode. For each AGRIS URI, the computation of combinations required 1.89 seconds on average. The execution time range was between 1.4 and 3.02 seconds for each AGRIS URI. Differences depended on three aspects:

- the number of AGROVOC URIs for a given AGRIS URI, which affects the response time of the AGRIS SPARQL endpoint. On average, an AGRIS resource contained 6 AGROVOC URIs;
- the specificity of an AGROVOC concept; broader concepts are associated to many web URLs, so they affect the response time of the Crawler Database SPARQL endpoint and
- network speed and delays.

It is important to note that this is a background, offline process that runs periodically to keep the database of recommendations up to date. End users querying AGRIS resources are served from this database, and are insulated from the processing time of the recommendation system. In this context, the aforementioned response times are perfectly acceptable as they allow for very frequent (daily or even more frequent) updates using moderate computational resources.

In a second experiment we ran the recommender system in the “individual” mode. The algorithm was executed on the same resources as for the previous experiment. For each AGRIS URI, the recommender system needed 2.3 seconds on average. Thus, using the recommender system as intermediate layer reduced the execution time of around 0.4 seconds for each AGRIS URI.

## The AGRIS front-end

**Figure 6.**
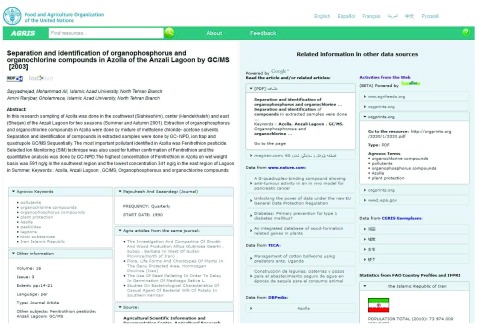
An AGRIS mashup page displaying an AGRIS bibliographic record with related information from external data sources.

The creation of an AGRIS mashup page (an example id provided in Figure 6) is a dynamic and sensitive process, which is made possible by the usage of AGROVOC as the backbone of the system. In fact, AGRIS records come with AGROVOC URIs and, relying on AGROVOC formal alignments to many thesauri, it is possible to query publicly accessible web services or SRARQL endpoints to provide access to resources indexed with various thesauri. Thus, when the user selects a publication from the AGRIS database, the system can display related information on the same topic. External data sources are identified based on the content, the relevancy to the AGRIS domain, and the information provider [ Celli *et al.*, 2015].

### A new data source: related resources crawled from the web

This paper presented a set of components that implement a process of discovering web resources related to AGRIS records in order to enrich the user experience in AGRIS mashup pages. We also ran experiments with the SemaGrow Stack as a backend component that can easily extend the data sources federated by the AGRIS mashup pages. These experiments led directly to the addition of a new data source to the AGRIS mashup pages: the dataset of related resources crawled from the web.

The creation of this new data source required setting up a process that used numerous components. We implemented an automatic process that makes use of a web crawler to discover web resources, and relies on AgroTagger to annotate discovered URLs with AGROVOC URIs. Then, in order to compute meaningful combinations between the AGRIS database and the Crawler Database, we implemented a recommender system to define the relevance of web resources. The output of this process is a widget in the AGRIS mashup pages that, reading the content of the Recommender Database (that is continuously updated by an offline process), can display relevant resources from the web. In this way, we believe that AGRIS users may find relevant data that assists them in working with agricultural issues and food security.

**Figure 7.**
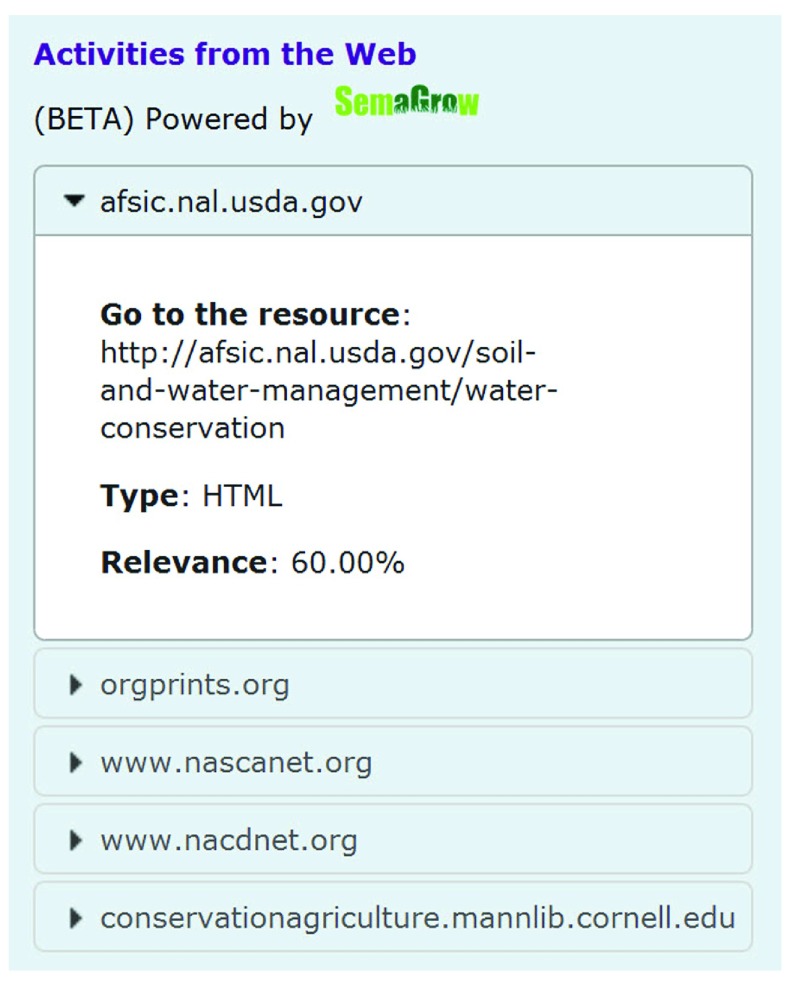
A widget displaying related resources from the web.

### Analysis of relevance

In this section we describe an evaluation study conducted on a benchmark sample of AGRIS articles, in order to determine the relevance of resources contained in the widget of Figure 7. We used a set of 10 AGRIS articles in the domain of fisheries and the relevance was evaluated by domain experts. Criteria to determine the test set were:

- AGRIS articles must be in the domain of fisheries (this is because we could rely on experts in the fisheries area). To cope with this requirement, we ran a query to the AGRIS Solr index, which is the index used by the website to allow end users to look for articles available in AGRIS; we used a Boolean “OR” query based on around 100 terms in the fisheries domain (including general keywords like “Fish”, “Fisheries”, “aquaculture”, but also more specific concepts like “Lumpfish”, “John dory”, “Sea bass”, “Whitefish”, “Weevers”, “Dolly varden” and “Carp”).
- AGRIS articles must be indexed with a number of AGROVOC keywords ranging from 4 to 8. In fact, as discussed in the “Similarity Index” section, while AgroTagger assigns 10 keywords to a crawled web resource, AGRIS record can contain on average 6 AGROVOC terms, ranging from 0 to 14. In our analysis we wanted to exclude extreme situations. We are obliged to make a consideration; there are two main strategies when a cataloguer indexes some articles: the cataloguer may use very few specific terms, or a lot of terms including also more general ones. Choosing to avoid extreme situations, we tried to balance between AGRIS records with too many general terms and records with very few specific terms. The mean of AGROVOC keywords per record in the AGRIS database was used as a pivot for our decision.
- 10 AGRIS articles were randomly selected by the subset of AGRIS articles meeting the previous two requirements.

Evaluating 10 AGRIS records means manually evaluating 50 recommendations coming from the recommender system. In fact, for each AGRIS record we recommend 5 web crawled resources in the widget “Activities from the web”. The evaluation followed a precise workflow:

- for each AGRIS article in the test set, we had to understand the topic of the article, considering the title, the abstract and the AGROVOC keywords available.
- for each recommended web resource in the widget “Activities from the web”, we identified if it was relevant to the AGRIS article or not. We paid special attention to the Similarity Score and to the level of granularity of AGROVOC keywords assigned to the AGRIS article.

Recommended web resources could have been identified as “not relevant” in some specific cases:

- they were considered generic news/publication pages, too vague to be relevant;
- the resource was geographically relevant, but the specific matter was completely different;
- the resource was related to a completely different subject;
- the resource was related to a too generic subject; this case was mainly due to the lack of specificity of AGROVOC keywords attached to an AGRIS article.

An important remark is that recommendations about the same specific topic of the AGRIS article were considered relevant even if they referred to a different geographical region. Moreover, we found one recommendation pointing to a web resource no more available (HTTP 404 response code). As an example, consider the AGRIS article “ *Monitoring and Surveing Fingerling Releasing (Kutum Bream and Pike Pearch) in Quality and Quantity Point of View*”, indexed with 6 AGROVOC keywords. One of the keywords is “fisheries”, which is may be too generic; in fact, the fourth recommendation (having a Similarity Score of 50%) is not relevant to the article because, even if it is geographically relevant, it talks about “fisheries”, while the AGRIS article is about “aquaculture techniques”. Note that the first three recommendations are all relevant (i.e. the precision is 0.6) and they present a Similarity Score between 50% and 66.67%.

**Figure 8.**
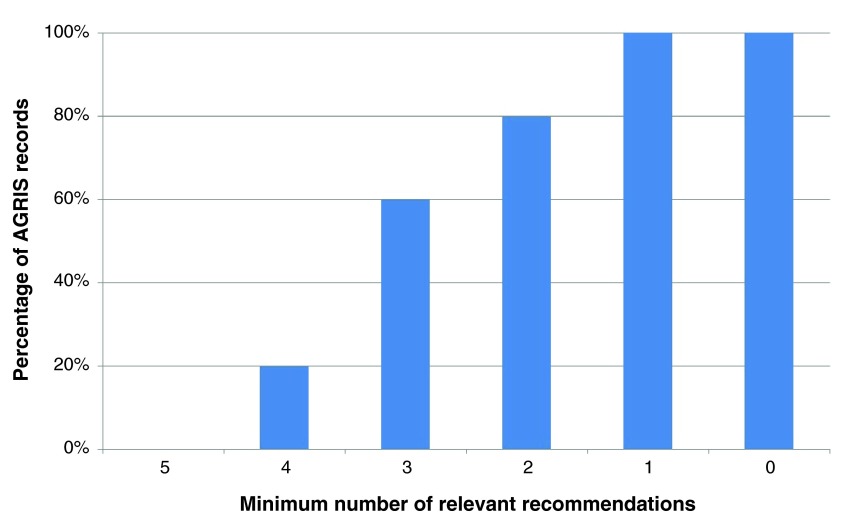
Cumulative distribution (percentage) of AGRIS records over the number of relevant recommendations.

Figure 8 displays the percentage of AGRIS records in the benchmark sample with a minimum number of relevant recommended resources; the histogram points out that at least one recommended resource per AGRIS article was considered relevant. The analysis of results showed a general precision of 0.52 (26 relevant resources over 50 analysed recommendations). For each AGRIS record in the test set, precision ranged from 0.2 to 0.8. 39% of recommendations considered as “not relevant” were too vague or related to a very general subject. Thus, some improvements to the algorithm of the recommender system can be made to get a better precision. The analysis also indicated that a Similarity Score bigger than 50% showed an acceptable level of precision: 27 recommendations had a Similarity Score greater than or equal to 50%, but one of them pointed to a non-existing web page; 18 recommendations were considered relevant, with a precision of 0.66 (0.69 if we exclude the recommendation no more available).

An interesting example can demonstrate how the algorithm implemented by the recommender system can be improved by matching AGROVOC terms of the AGRIS article with the titles and descriptions of crawled web resources. Let’s consider an extreme situation, i.e. an AGRIS article with many AGROVOC keywords, including also more generic ones. The article “ *Water Conservation of Qanat, using Optimum use of Water in Unused Seasons (Case study: Dehraz Qanat of Sabzevar)*” exposes some criteria to optimize the use of water carried by qanats (a qanat is an underground channel to transport water from an aquifer under a hill, especially for irrigation of hot and arid environments). Thus, as it is highlighted by the title, the article is mainly about “water conservation”. It has been indexed with eleven AGROVOC terms, including water management, soil sciences, soil conservation, and water conservation. The recommender system suggests five web resources related to this article, with a similarity score ranging between 42% and 60%. The first suggestion is a web page of the USDATA.GOV website ( http://afsic.nal.usda.gov/soil-and-water-management/water-conservation); the score is 60% and the resource is completely related with the article, since it contains other resources about “water conservation” coming from USA universities and other agencies. Matching AGROVOC terms of the AGRIS article with the title of this resource would have further increased the Similarity Score, since the title itself is about “water conservation”. Regarding the remaining four suggestions, the example shows how a similarity score of 42% does not predict good results. For instance, the web page of Cornell University ( http://conservationagriculture.mannlib.cornell.edu/pages/resources/photosvideos.html) is relevant to the article, since it contains – among other things – some Power Point presentations about “water management” and “soil management”. Conversely, the other resources with the same similarity score of 42% are irrelevant. The improved algorithm (matching AGROVOC terms of the AGRIS article with the titles and descriptions of crawled web resources) would suggest the XML feeds from EPRINTS about “agricultural water management”, and the PDF document entitled “ *Agricultural Perspectives on Water Resource Management in the Americas*”, both of which are quite relevant and yet missing from the current list. In this case, the improved algorithm would increase the precision from 0.4 to 0.8.

## Conclusions

The web contains much latent knowledge, especially when that knowledge is expressed as unstructured and poorly categorized full-text content. This paper describes a proposed solution to discover such knowledge making use of modified open source software (Nutch and Maui) together with the SemaGrow Stack and a custom recommender in order to enrich the relevance of AGRIS bibliographic resources and hence the AGRIS web portal mashup.

The adoption of the SemaGrow Stack as a backend facilitated the development of a recommender engine as it was possible to implement the system without requiring any prior knowledge of the specifics of the datasets that are combined with the AGRIS database. In this manner, the system can be re-used with any dataset using AGROVOC (or any terminology aligned to AGROVOC) to describe websites, experiments, software, or any resources relevant to agriculture. We are now able to show to AGRIS users any relevant content extracted from the web, something possible thanks to the adoption of semantic web technologies. The entire process will continue to be extended and improved as experiments continue: AgroTagger and the recommender system will be tuned to guarantee better precision in the computation of recommendations (for instance, trying to match AGROVOC keywords with titles of target resources and experimenting with alternative algorithms), while the SemaGrow Stack will be optimized and used to federate additional data sources.

Furthermore, as previously mentioned, training AgroTagger with a different thesaurus allows to apply the entire process to different research domains, and not only to the AGRIS website.

## Software availability

Latest source code (AgroTagger): https://github.com/fcproj/agrotagger

Latest source code (recommender system): https://github.com/fcproj/recommender

Archived source code at the time of publication (AgroTagger): http://dx.doi.org/10.5281/zenodo.20777 ( Celli, 2015a).

Archived source code at the time of publication (recommender system): http://dx.doi.org/10.5281/zenodo.20775 ( Celli, 2015b).

License: CC BY 4.0 http://creativecommons.org/licenses/by/4.0/

## References

[ref-1] Anibaldi S Jaques Y Celli F: Migrating bibliographic datasets to the Semantic web: The AGRIS case. *Semantic Web.* 2015;6(2):113–120. 10.3233/sw-130128

[ref-2] Berendt B Hotho A Mladenic D: A roadmap for web mining: from web to semantic web. *Web Mining: From Web to Semantic Web*. vol. 3209 of Lecture Notes in Computer Science, Springer, Berlin, Germany. 2004;3209:1–22. 10.1007/978-3-540-30123-3_1

[ref-3] Caracciolo C Morshed A Stellato A: Thesaurus Maintenance, Alignment and Publication as Linked Data: the AGROOVOC use case. In *Proceedings of the 5th Intl Conference on Metadata and Semantic Research Proceedings*, Izmir, Turkey.2011;240:489–499. 10.1007/978-3-642-24731-6_48

[ref-4] Celli F Malapela T Wegner K: AGRIS: providing access to agricultural research data exploiting open data on the web [v1; ref status: approved 1 http://f1000r.es/599]. *F1000Res.* 2015;4:110. 10.12688/f1000research.6354.1 26339471 PMC4544375

[ref-5] Celli F: agrotagger: crawler_agrotagger_1_2_5_DOI. *Zenodo.* 2015a Data Source

[ref-6] Celli F: recommender: agris_recommender_system_1_3_2_DOI. *Zenodo.* 2015b Data Source

[ref-7] Charalambidis A Troumpoukis A Konstantopoulos S: SemaGrow: Optimizing federated SPARQL queries. In *Proceedings of the 11th International Conference on Semantic Systems (SEMANTiCS 2015)*, 15–18 September, Vienna, Austria.2015;121–128. 10.1145/2814864.2814886

[ref-8] Devi RS Manjula D Siddharth RK: An Efficient Approach for Web Indexing of Big Data through Hyperlinks in Web Crawling. *ScientificWorldJournal.* 2015;2015:739286. 10.1155/2015/739286 26137592 PMC4475586

[ref-9] Kouper I: Science blogs and public engagement with science: Practices, challenges and opportunities. *Journal of Science Communication.* 2010 Reference Source

[ref-10] Liakos P Ntoulas A Labrinidis A: Focused crawling for the hidden web. In *Proceedings of the World Wide Web 2015 Conference Proceedings*, Florence, Italy.2015 10.1007/s11280-015-0349-x

[ref-11] Medelyan O Witten IH: Domain independent automatic keyphrase indexing with small training sets. *J Am Soc Inf Sci Tec.* 2008;59(7):1026–1040. 10.1002/asi.20790

[ref-12] Shema H Bar-Ilan J Thelwall M: Research blogs and the discussion of scholarly information. *PLoS One.* 2012;7(5):e35869. 10.1371/journal.pone.0035869 22606239 PMC3350512

[ref-13] Soulemane M Rafiuzzaman M Mahmud H: Crawling the Hidden web Approach to Dynamic web Indexing. *Int J Comput Appl.* 2012;55(1):7–15. 10.5120/8717-7290

[ref-14] Zoulis N Mavroudi E Lykoura A: Workload-Aware Self-Tuning Histograms of String Data. In *Proceedings of the 26th DEXA Conference (DEXA 2015)*, 1–4 September, Valencia, Spain.2015;9261:285–299. 10.1007/978-3-319-22849-5_20

