# Supplementary material for: Discovering, Indexing and Interlinking Information Resources
Source: F1000Res. 2015 Nov 17;4:432. Originally published 2015 Jul 30. [Version 2] doi: 10.12688/f1000research.6848.2 (PMC4722689; doi:10.12688/f1000research.6848.2)
Supplement: Peer review history

# Peer review: Referee response for version 2

**DOI:** 10.5256/f1000research.7931.r11452

**Published:** 2015-12-08

**Authors:** Leonidas Papachristopoulos

**Affiliations:** 1 Department of Archives, Library Science and Museology, Ionian University, Corfu, Greece

Ι have to admit that the authors of the paper improved significantly the quality of their work. I am very glad to see that the performance oriented issues have been adequately analysed and presented. The paper presents in detail the whole workflow of the experiments and the quality of its outcomes without leaving any doubts on the capabilities of AGRIS.

I have read this submission. I believe that I have an appropriate level of expertise to confirm that it is of an acceptable scientific standard.

# Peer review: Referee response for version 2

**DOI:** 10.5256/f1000research.7931.r11243

**Published:** 2015-12-01

**Authors:** Paolo Missier

**Affiliations:** 1 School of Computing Science, Newcastle University, Newcastle upon Tyne, UK

The manuscript is improved, I appreciate the authors' response to my comments (and those of the other referees).  I have no real objections, however as I read the "checklist" for referees, may I suggest:

- the title may be too generic for the specific contribution provided?

Also, I know complete reproducibility is a Nirvana state, hardly ever attained in practice, but I appreciate the SW source code is available. I haven't checked but are you confident that anyone other than the authors can do anything useful with it?

I have read this submission. I believe that I have an appropriate level of expertise to confirm that it is of an acceptable scientific standard.

# Response

**Published:** 2015-12-02

**Authors:** Fabrizio Celli

**Affiliations:** Food and Agriculture Organization of the United Nations, Italy

We want to thank again Dr. Missier for his comments. As far as the title concerns, we decided to make it very general, since the workflow and components that we describe in the paper can be adapted to all domains. It is true that our use case (i.e. AGRIS) refers to the domain of agricultural science and technology. Anyway, as we discussed in the paper, what makes the workflow domain-specific is the AgroTagger; if someone trains such tool with a SKOS thesaurus in a different domain, he/she can apply the workflow to the new use case. It is also true that there are other restrictions; for instance, you should use a thesaurus available as SKOS; you should be prepared to work with RDF and SPARQL, since the output of the recommender system is a set of triples, as well as its input is a set of SPARQL endpoints. But the entire workflow is applicable to the entire web.

About the code, it is very easy to be learned and used. Documentation is provided within the code, and the software components are highly configurable (you simply need to change a configuration file and then to execute a JAVA class). The only difficult part is training the AgroTagger with a new thesaurus; in fact, it requires to build a new MAUI model, which needs a medium-large set of documents already indexed with the specific thesaurus by humans.

# Peer review: Referee response for version 2

**DOI:** 10.5256/f1000research.7931.r11244

**Published:** 2015-11-23

**Authors:** Kei Kurakawa

**Affiliations:** 1 National Institute of Informatics, Tokyo, Japan

The paper has been modified to take my suggestions into consideration. The authors strongly took care with a more delicate explanation for the quality of proposed functionality, i.e., recommendations of web resources for an AGRIS article. They also revised the whole description of their thoughts on the paper in an acceptable way. This paper, as a software tool article, is acceptable to be indexed.

I have read this submission. I believe that I have an appropriate level of expertise to confirm that it is of an acceptable scientific standard.

# Peer review: Referee response for version 1

**DOI:** 10.5256/f1000research.7363.r10769

**Published:** 2015-10-12

**Authors:** Leonidas Papachristopoulos

**Affiliations:** 1 Department of Archives, Library Science and Museology, Ionian University, Corfu, Greece

Current study entitled "Discovering, Indexing and Interlinking Information Resources" consists of a technical evaluation of an enriching system attached to an agricultural bibliographic database called AGRIS. I am not sure about the novelty of the system that is presented but I can admit that is an interesting work which does not pose any reading difficulties to the reader, even to those who are not so familiar. Lately, recommending systems have been set on the center of the research activity and the specific work is following the research trends of the area.

In my opinion there are some gaps in the study. For example on the description of the Agrotagger crawler researchers say:

“Regarding accuracy, a recent test in which AgroTagger results were analyzed by professional indexers showed an accuracy of approximately 80%; in 20% of the cases the results were too broad. In brief, the accuracy measurement was carried out by domain experts actively involved in the development of the AGROVOC vocabulary based on manual annotations of a test sample from the AGRIS database”

It is not clear to me if the aforementioned test has been held in the context of another previous study or it covers the specific study. If it has been held in the past a reference is necessary. Otherwise researchers need to provide further details.  Additionally I think that authors should provide more information about accuracy tests. In my opinion the paper describes adequately the design and implementation but needs further enrichment regarding evaluation data.

Authors should consider papers restructuring as in many cases I get lost between implementation and evaluation points.

Some minor syntax comments are:

In Page 1 *:* *“AGRIS database, together with other related resources extracted from other preselected datasets*”… “together with” should be changed to “combined with”.

In Page 1: " *Nowadays, scientists and researchers publish their results not only in journals or at conferences, but also via web 2.0 tools and other media [ Kouper, 2010;* *Shema et al., 2012] in order to efficiently and broadly communicate their outcomes, [a technique that also helps scientific research reach the general public, since newspapers, magazines and science blogs are often the quickest way to reach people informally]."* For better understanding the sentence should be split into two. From the point “a technique’ should start another sentence.

In Page 1 *: "Blogs and other websites may also contain a corpus of ongoing …"* “Corpus” should be changed into “corpora”

In Page 2: *"Adopting Semantic Web and LOD best practices and technologies, AGROVOC vocabulary items have been assigned URIs, organized into a SKOS-XL concept scheme ( http://www.w3.org/TR/skos-reference/skos-xl.html), and served both as Linked Open data and via SPARQL endpoint ( http://www.w3.org/TR/sparql11-overview)."* Re-write the sentence for better understanding.

I have read this submission. I believe that I have an appropriate level of expertise to confirm that it is of an acceptable scientific standard, however I have significant reservations, as outlined above.

# Response

**Published:** 2015-11-03

**Authors:** Fabrizio Celli

**Affiliations:** Food and Agriculture Organization of the United Nations, Italy

Really thanks for your comments. As you suggested, the “AgroTagger” section was improved. We added a reference to a paper including details about the MAUI training set. We also added a more precise description of the test sample used to measure accuracy of AgroTagger. In addition to that, evaluation of performance was moved to a separate section, together with further details as the comparison between the execution time in the individual and federated modes. Moreover, we improved the evaluation of relevance of recommendations:  we ran an analysis of relevance on a test set of resources in the domain of fisheries, computing precision and providing other comments. We hope that, based on these modifications, the referee can now fully approve our article.

# Peer review: Referee response for version 1

**DOI:** 10.5256/f1000research.7363.r10291

**Published:** 2015-09-28

**Authors:** Kei Kurakawa

**Affiliations:** 1 National Institute of Informatics, Tokyo, Japan

This paper proposed a functional enhancement for the agricultural journal article bibliographic database search, AGRIS, whose bibliographic page has additional web resources information relevant to its content. The web resources are automatically crawled by a customized Nutch web crawler, and labeled with some agricultural index terms, i.e. AGROVOC by a kind of machine learning module, the AgroTagger classifier. Since AGRIS bibliographies are already labeled with AGROVOC, the paper proposed a similarity measure between AGRIS bibliographies and web resources based on the number of common and originally labeled AGROVOC terms on them. AGRIS bibliographies and web resources are stored in SPARQL servers, so that the SemaGrow stack is used to easily send queries to them to calculate the similarity scores. The recommender system provides at most top 20 relevant web resources for an AGRIS bibliography by the calculation, which is serialized on RDF/XML.

I recognize that the whole framework to construct the new functionality is valuable, but the paper is not sufficient with computational performance evaluation on the framework and quality evaluation for the linkages between bibliographies and web resources in an approved scientific method.

Below are the suggestions for minor improvements.

In page 3:

In the “Crawling and indexing the web” section, the author describes “In the section 2.1 and 2.2” in the leading paragraph. The section numbers might not be forbidden to point to sections of this article.

In page 4:

In the left column of the “web crawler” sub-section, the author shows a snap shot of the output file of a Nutch web clawer. Please describe the structure of the file. I would like to know what some tags mean to construct the structure. Why is a blank URL listed in the example?

In the left column of the “AggroTagger classifier” sub-section, the author mentions the quality of tagging by AgroTagger such as “AgroTagger results were analyzed by professional indexers showed an accuracy of approximately 80%; in 20% of the cases the results were too broad.” I would like to know more precise descriptions of the quality of tagging. How large is the training set? How did the author construct the training set? Please describe the training set. In addition, the test set for tagging and examining its accuracy should be described.

In the “AggroTagger classifier” sub-section, the figure 3 and 4 should be explicitly referenced. In the current article, those figures are not referenced in any sentences.

In the first phrase of “The SemaGrow Stack” section, the author refers “the data-intensive and inter-disciplinary Science of 2020”. Does “2020” means “Horizon 2020”? Or, does it refer to another funding framework? Science of 2020? If it is another funding framework, please cite a relevant web site for it. Please make sure what it is.

In page 6:

In the “recommender system” section, the second paragraph of “The recommender system can make use of … add further endpoints to the process”, the figure 5 should be explicitly referenced. In addition, for good understanding of reconfigurable structures consisting of the “federated” and the “individual”, the author had better depict both structures in the figure 5.

In page 7:

In the right column, the author describes “the overall custom algorithm” as a list form. For better understanding, this algorithm should be represented in a pseudo-programming code form.

In the right column, the author mentions some experimental conditions and results of the number of AGRIS URIs and the average execution time. In addition, how many AGROVOC URIs are prepared for AGRIS URIs or Web URLs in this experiment? How long does it take for all processing? Please figure out the time of processing in the “individual” mode and the “federated” mode. Which is better from the viewpoint of computation?

In page 8:

In the right column, line 13, “factor K” should be changed to the lower case “factor k”.

In the last similarity formula, “braces ( )” should be written in the proper place.

In table 2, “Real Score” might be “ Revised Score”?

In page 9:

In the right column, the section “AGRIS front-end”, please indicate explicitly figure 6 in a sentence.

I have read this submission. I believe that I have an appropriate level of expertise to confirm that it is of an acceptable scientific standard, however I have significant reservations, as outlined above.

# Response

**Published:** 2015-11-03

**Authors:** Fabrizio Celli

**Affiliations:** Food and Agriculture Organization of the United Nations, Italy

We would really like to thank Dr. Kei Kurakawa for this useful review. The paper was improved following his comments. In particular:

1. We separated the performance experiments from the deployment section. Performance experiments were performed again using a bigger test set and we compared execution time in the “individual” mode with the execution time in the “federated” mode.
2. We ran an analysis of relevance on a test set of resources in the domain of fisheries. We were able to compute precision of linkages, adding some additional details.
3. The “web crawler” section was revised: we described the structure of the output file, which is generated using the “readseg –dump” command line tool provided by Apache Nutch. The blank URL listed in the example was meaningless, so we removed it.
4. The “AgroTagger” section was revised too, with the addition of a more precise description of the test sample used to measure accuracy. Then, the paper including the work of training MAUI was cited.
5. In the “SemaGrow Stack” section, the reference to the “Science of 2020” was removed, since it was misleading. In fact, it is part of SemaGrow proposal description, so it does not explicitly refer to Horizon 2020 or to any other framework, but to a general view of the world in 2020.
6. All minor improvements suggested by the reviewer were implemented.

We hope that, based on these modifications, the referee can now fully approve our article.

# Peer review: Referee response for version 1

**DOI:** 10.5256/f1000research.7363.r9730

**Published:** 2015-08-24

**Authors:** Paolo Missier

**Affiliations:** 1 School of Computing Science, Newcastle University, Newcastle upon Tyne, UK

Overall, I found the article interesting and easy to read. In line with the F1000 reviewers' guidelines, I am going to comment "on the quality of the research and whether the article is scientifically sound (i.e. whether the work has been well designed, executed and discussed)", rather than on novelty, also making suggestions for improvements in the presentation.

The work partly builds on prior established tools designed for the AGRIS system, mainly the AGROVOC thesaurus, as well as methods for federating SPARQL queries over heterogeneous resources (the SemaGRow stack).

The original research contribution consists mainly of a focused crawler that looks for web resources that are relevant to the AGRIS community, the AgroTagger annotation tool, which annotates those web resources with semantic terms from the AGROVOC thesaurus, and a recommender tool that uses the annotated database to suggest web resources that are likely to be relevant to a given AGRIS bibliographic item. 

The annotation model is fine, however the evaluation part of the paper is weak, especially with regards to recommendation. This is where I think the authors have an obligation to report on the accuracy (ie F-scores) of their method on a benchmark of test cases, especially since substantial effort is spent in the paper to explain the similarity score used by the recommender. This is completely missing, however, the only evidence of the method at work being an example towards the end.  For an experimental paper, this is hardly acceptable. So my main substantial recommendation is to strengthen the evaluation section (this may appear in other articles cited in the references, however that was not immediately clear to me).

On matters of form and presentation, I found that the methods, including the recommender algorithm, are very simple (by the authors' own admission) and thus in my opinion the exposition can be greatly abbreviated. For example, fig. 2 and 3 are not very  informative and could be removed. Also the lengthy RDF fragments are not very significant (but if you need them, please switch to n3 notation!), the annotation model of fig 4 is very simple (essentially, just a dcterms:subject), the 6-points algorithm is a straightforward intersection operation and has already been described in the text, the queries on pg 7 are fairly simple, etc.

Further minor presentation suggestions:

- You may not need to build up the similarity score from naive to final, possibly just describe the final version?
- There is a mix of design and implementation / performance considerations, these may be best kept separate.
- A new section header may be missing after the first paragaph of "agris front end"?

I have read this submission. I believe that I have an appropriate level of expertise to confirm that it is of an acceptable scientific standard, however I have significant reservations, as outlined above.

# Response

**Published:** 2015-11-03

**Authors:** Fabrizio Celli

**Affiliations:** Food and Agriculture Organization of the United Nations, Italy

We would really like to thank Dr. Paolo Missier for his useful comments.

We ran an analysis of relevance on a test set of resources in the domain of fisheries. We were able to compute precision, but not recall. In fact, recall is mainly related to the web crawling, and it is not easy to estimate it. In addition to that, in the context of the AGRIS website it is not strongly important: we are more interested in a good precision, especially because we display only 5 recommendations per AGRIS resource.

We separated the performance experiments from the deployment section, adding further details. Performance experiments were performed again using a bigger test set and we compared execution time in the “individual” mode with the execution time in the “federated” mode.

We abbreviated a bit the exposition (for instance, we removed the six-points algorithm and the RDF fragment related to the output of the recommender system), but we maintained the discussion about the flow of thoughts leading from the naïve similarity score to the final one because we thought it helps in justifying some parameters available in the final formula.

We hope that, based on these modifications, the referee can now fully approve our article.
